# Female physician and pregnancy- effect of the amended German maternity protection act on female doctors’ careers

**DOI:** 10.1515/iss-2022-0024

**Published:** 2023-03-31

**Authors:** Barbara Puhahn-Schmeiser, Eva K. Hennel, Christiane Gross, Heike Raestrup, Astrid Bühren, Mandy Mangler

**Affiliations:** Department of Neurosurgery, University Medical Center, Albert-Ludwigs-University, Freiburg im Breisgau, Germany; German Medical Women’s Association, Berlin, Germany; Department of Medical Education, University of Bern, Bern, Switzerland; MVZ Limburg, Limburg an der Lahn, Germany; Department Obstetrics and Gynecology, Vivantes, Auguste-Viktoria-Klinikum, Berlin, Germany

**Keywords:** career, female medical student, female physician, maternity protection act, pregnancy, surgery

## Abstract

**Objectives:**

In Germany, the 2018 amended Maternity Protection Act frequently leads to fundamental restrictions for female physicians, especially surgeons, and now even also for students impeding the progress of their careers. Our goal was to assess the current situation for pregnant female physicians and students, respectively, and their perspective on this amendment regarding their career path.

**Methods:**

A nationwide survey was conducted in Germany from December 2020 to February 2021. The questionnaire included 790 female physicians and students who were pregnant after the inception of the amended Act. Those women pregnant after the beginning of the corona pandemic were excluded.

**Results:**

The survey revealed that two thirds of female physicians worked a maximum of 50% in their previous professional activity as soon as they reported pregnancy. Amongst medical students this amounted up to 72%. 18% of the female physicians and 17% of the female medical students respectively could not follow the sense of these restrictions. 44% of female medical physicians and 33% of female students felt their career impeded. This led up to 43% amongst female medical doctors and 53% amongst female medical students, respectively, who were concerned to announce their pregnancy. As a consequence, pregnancies were reported at 12 weeks in female physicians compared to 19 weeks in medical students.

**Conclusions:**

Analyses of the current survey revealed that a relevant number of female physicians and medical students felt impeded in their career path through the application of the amended Maternity Act.

## Introduction

In 2018, Germany amended its Maternity Protection Act, which was initially meant to reinforce female participation in professional work. This amendment has led to restrained employment of pregnant female physicians for direct patient work and even for pregnant medical students. The reason for this is the widely interpretable legal text impeding employers to further employ pregnant doctors, despite restructuring working conditions as well as despite strict adherence to protective measures. As a consequence, pregnant doctors are assigned for tasks, not or predominantly not relevant for their further medical specialist training or career.

In order to investigate the current situation for pregnant female physicians and their perspective on this amendment, we performed a nation-wide survey amongst female doctors of all medical specialties as well as medical students.

## Methods

This study comprises data from an online survey amongst female students and doctors, distributed through the German Medical Women’s Association and additionally shared by respondents. It was performed between December 2020 and February 2021 under the leadership of the German Medical Women`s Association (Deutscher Ärztinnenbund, DÄB). The questionnaire included sociodemographic data as well as information about career development before and during pregnancy. The cohort of respondents comprises 790 female students and doctors of different specialties from the whole of Germany who were pregnant after the inception of the amended Act in January 2018. In order not to have confounders caused by regulations due to the corona pandemic, women pregnant in March 2020 or later were not included.

Statistical analyses were carried out in SPSS, version 22. Data were analyzed based on descriptive methods.

## Results

### Sociodemographic and professional data of the cohort

**Figure 1:**
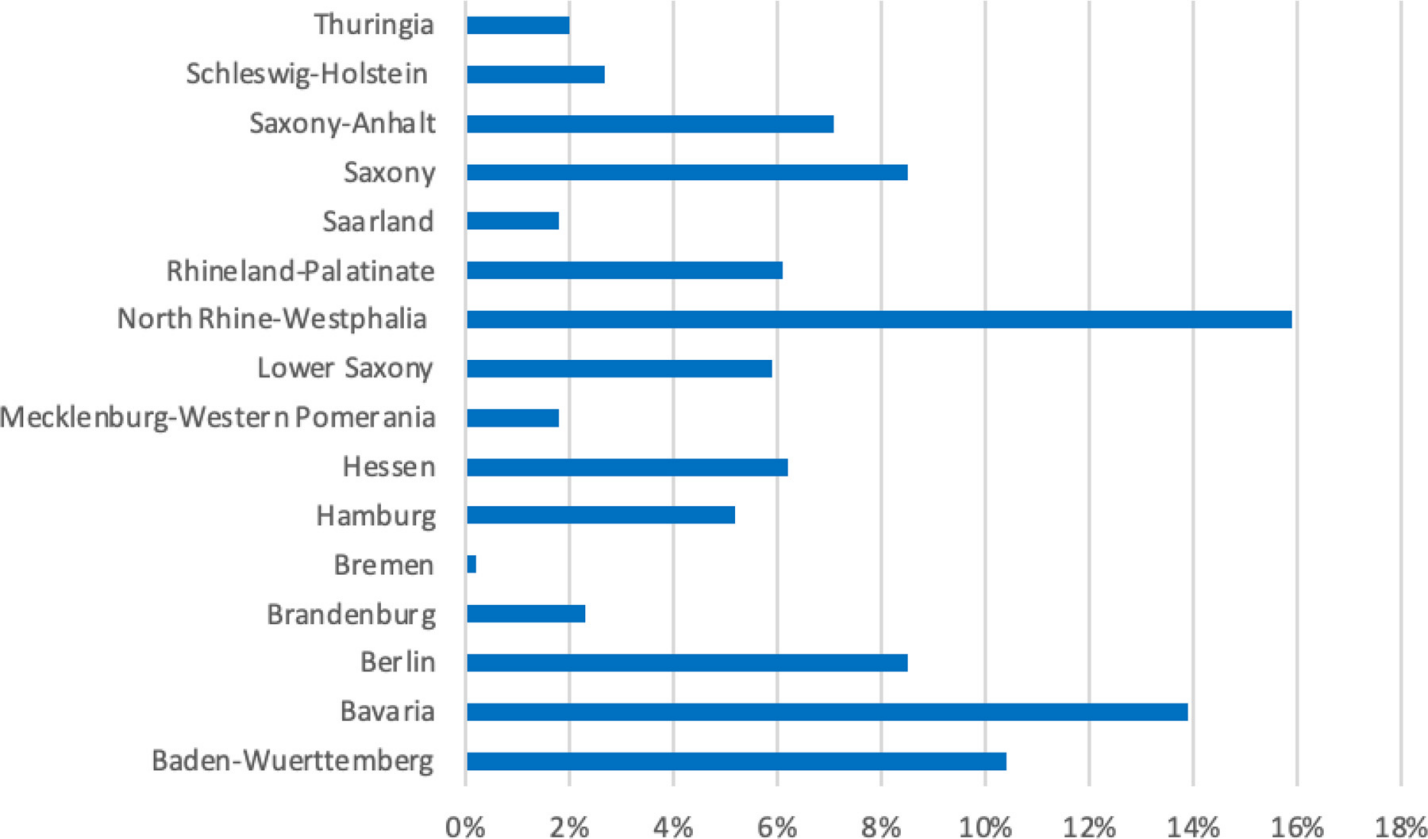
Distribution of participants regarding German federal states. Distribution of participating female students and physicians in different federal states of Germany.

Sociodemographic and professional data are presented in Table 1. The cohort comprises 790 female students and physicians. Of those evaluated 10% were students, 89% were doctors working in patient-near specialties and 2% were doctors working in patient-remote specialties. Physicians who participated in this interview were predominately working in non-university hospitals (56%), followed by university hospitals (26%) and out-patient clinics (8.5%). The geographical distribution of the participants was spread nationwide (Figure 1), with a percentage of 10% from Baden–Wuerttemberg, 14% from Bavaria, 15% from North Rhine-Westphalia and 10% from Saxony. Other federal states were also represented, yet to a lower extent with a percentage of under 10% respectively (Figure 1).

**Table 1:** Sociodemographic and professional data of the cohort.

		n, %
**Total**		790 (100.0)
**Participants**	Students	55 (7.0)
	Practical year students	28 (3.5)
	Interns	526 (66.6)
	Specialists	117 (14.8)
	Senior physicians	46 (5.8)
	Others	18 (2.3)
**Employement**	University hospital	208 (26.3)
	Non-university hospital	449 (56.8)
	Medical practice	63 (8.0)
	Self-employed in medical practice	4 (0.5)
	Others	17 (2.2)
	Currently not employed	49 (6.2)

**Figure 2:**
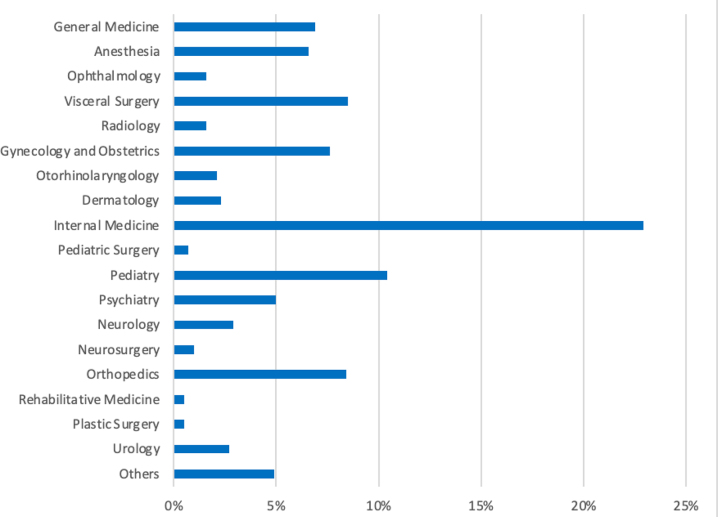
Distribution of participants regarding medical specialty. Distribution of participating female students and physicians in different medical specialties.

Female doctors from many different medical specialties participated (Figure 2). Of those, internal specialists were represented in 21% of the cases, surgeons in 16%, pediatricians in 9%, and anesthesiologists in 5%.

### Pregnancy-related data in the whole cohort

Pregnancy was reported to the employer on average in the 12th week of pregnancy. 43.9% of the participants stated they had concerns to report pregnancy. In 16.1% of these physicians, pregnancy announcement resulted in direct employment bans. Only 7.2% of the participants were able to continue working unaffectedly having been provided strict protective measures. One third of the participants (30.2%) had their professional activity reduced to 70% of their previous work load due to their pregnancy. The vast majority of pregnant doctors in this study (62.7%) had a radical change of daily work activities: 50% of their professional activity was altered and they were not able to continue working as before their pregnancies. Restrictions were found to be at least partly reasonable to 81.4% of the respondents, yet 18.6% found the restrictions unreasonable and 43.1% felt set back in their careers.

### Pregnancy-related data in the subgroup of female physicians

Amongst female doctors who had participated in the survey, pregnancy was reported to the employer in the 11th week. Whereas 56.8% of the participants did not have concerns to report pregnancy, 43.2% did so. In 16.7% of the interviewed, their pregnancy announcement resulted in a direct employment ban. Only 7.9% of the interviewed could completely continue their previous work under strict protective measures; 30.2% could only perform 70% of their previous professional activity and 61.3% of the participants could only perform 50% of their previous professional activity (50% restriction: 21.9%; 70% restriction: 20.7%; 100% restriction: 19.3%). Restrictions were found to be at least partly reasonable to 81.6% of the interviewed, yet 18.4% did not understand the sense of these restrictions. Whereas 55.8% of the interviewed did not see their career impeded, 44.2% felt to be hindered.

### Pregnancy-related data in the subgroup of female students

Subgroup analyses of exclusively female students consistently differed from female doctors. Amongst students, pregnancy was reported to university at a later stage as compared to the group of physicians. Female students chose to report pregnancy only in the 19th week of pregnancy. In the student cohort, 53.3% of pregnant women did have concerns to reveal their pregnancy. Only 2% of the interviewed students could continue their previous activities under strict protective measures, whereas 26% could perform 70% of their previous activities and 72% were restricted in their medical student activities over 50% (50% restriction: 36%; 70% restriction: 18%; 100% restriction: 18%). 82.8% found restrictions at least partly reasonable, whereas 17.2% did not understand the sense of these restrictions. 66.1% of the interviewed students did not see their career impeded, yet 33.9% felt to be hindered.

## Discussion

Since 1952, German female professionals can rely on a Maternity Protection Act, a landmark which sets the framework conditions for safe working under strict protective measures to ensure well-being for the mother and the unborn child. After several modifications over the years, this act has lastly been amended in 2017 and came into effect in January 2018 [1]. This amendment contained the redefinition of requirements for working hours and uninterrupted resting time as well as night, Sunday and holiday work. Besides, employers were obliged to perform a risk assessment in order to restructure the working place and conditions, respectively, aiming to prevent employment bans. In addition, violation of the employer’s obligation to carry out a risk assessment is even considered an administrative offense. For the first time, also students and trainees were included in this Act.

The fundamental idea of this Act was supposed to facilitate females to continue working under save conditions [2]. Yet, its practical implementation has rather further aggravated the situation of pregnant women, especially in the health care sector. It has led chief physicians and hospital managements to restrain further employment of pregnant doctors despite restructuring working conditions and despite strict adherence to protective measures. As a consequence, pregnant doctors are assigned for tasks outside their core profession such as paperwork only, which are not or predominantly not relevant for further medical specialist training. A significant number of medical staff even experience employment bans while pregnant. Most of the time, female doctors are not allowed to continue their work in the operating room or in other functional areas in medicine leading to the delay in the completion of their training. This career delay is a disadvantage and did not only affect female trainees but also specialists and senior physicians. Thus, in every stage of their career they were negatively affected by a law meaning to support them.

Female doctors are underrepresented in leadership positions especially in respect to the number of female students starting their medical career. Our analysis shows that the practical transfer of the new German Maternity Protection Act has severe consequences for pregnant doctors and medical students.

**Figure 3:**
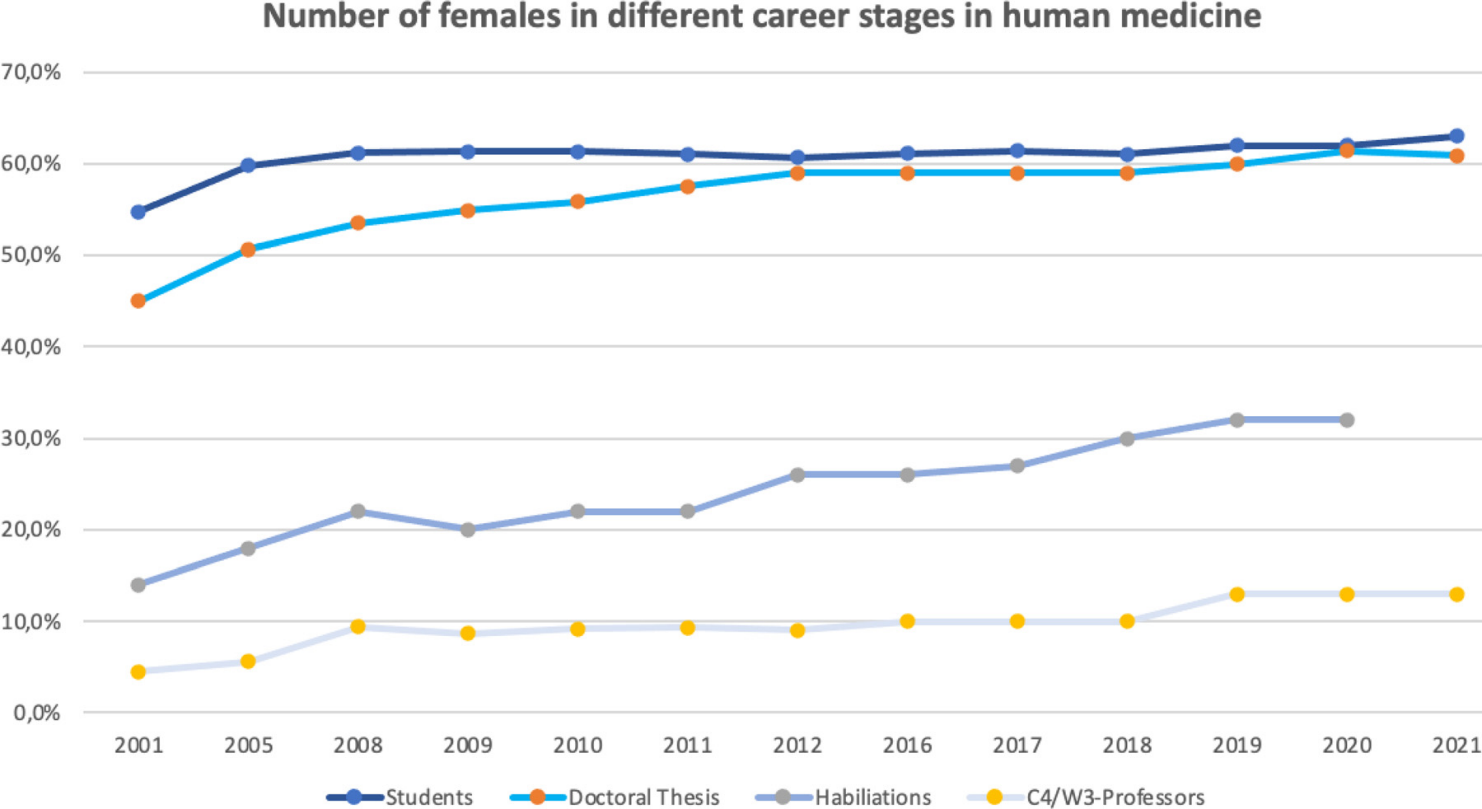
Number of females in different career stages in human medicine. Illustration of the process of female doctors’ participation in different career stages in human medicine (studies, doctoral thesis, habilitation, C4/W3 professorships) between 2001 until now.

Pervasive gender disparities exist in medicine regarding graduation, achievement of academic rank, and appointment to leadership positions [3], [4], [5]. Over the last years, the number of female medical students has steadily risen, exceeding the number of male students since 1999. Meanwhile two thirds of Germany’s medical students are female [6, 7]. During medical studies, gender distribution remains quite stable, even though a minor decline of female students is already evident. The number of doctoral thesis performed by female students are reported to be still higher than by male students [8, 9] but when looking at the career path between thesis and habilitation, females show a drastic decline. The number of female habilitations only reaches 32% of all habilitations [10]. Although, overall a minimal rise in the number of female habilitations can be observed over the years, the number of female doctors holding professorship in Germany’s university hospitals is stagnating, amounting to 13% according to a recently updated survey Medical Women on Top [5] (Figure 3). According to the Association of American Medical Colleges (AAMC) females in the USA account for 43% of medical school faculty but only 21% of department chairs and 19% of medical school deans [11, 12]. There seems to be a negative impact on female careers after medical school, especially between thesis and habilitation. Our study shows that one part of the explanation for this negative impact lies in the time when families are formed and women become pregnant. Yet, restraining female employment and career has the potential to further deteriorate the tense situation in the health care system due to the rising number of female medical students and female doctors respectively. Female doctors who care about patients’ well-being on a daily basis and also care for pregnant patients and their empowerment are deprived of their own right to decision making regarding their own health issues. As doctors are medically qualified people this is especially difficult to understand. Heteronomy of the female body permeates all structures of society and has to find an end to build a gender equal society.

Some clinics help themselves by finding individual solutions to support pregnant medical staff. They focus on self-empowerment of their pregnant colleagues thus encouraging them to keep on working and focusing on their careers in case of an uncomplicated pregnancy. Decisive constructive suggestions from the federal committee for Maternity Protection according to paragraph 30 of the amended act are expected. A further approach to this issue is a stronger link of maternity protection and occupational safety according to the current evaluation report of the federal government following the inception of the Maternity Act [2].

## Conclusions

The interpretation of the actual Maternity Protection Act leads to additional downtimes, mostly during specialty training but lately even already during medical studies. Considering the current lack of doctors and demographic development in Germany, enabling pregnant doctors to continue working under safe conditions should be thrived for if this is the wish of the pregnant woman in case of uncomplicated pregnancy.

## Supplementary Material

Supplementary Material

## References

[1] Bundesamt für Justiz Gesetz zum Schutz von Müttern bei der Arbeit, in der Ausbildung und im Studium 2017.

[2] D Bundestag Evaluationsbericht der Bundesregierung über die Auswirkungen des Gesetzes zum Schutz von Müttern bei der Arbeit, in der Ausbildung und im Studium 2022.

[3] Mousa M, Boyle J, Skouteris H, Mullins AK, Currie G, Riach K (2021). Advancing women in healthcare leadership: a systematic review and meta-synthesis of multi-sector evidence on organisational interventions. EClin Med.

[4] Kuhlmann E, Ovseiko PV, Kurmeyer C, Gutiérrez-Lobos K, Steinböck S, von Knorring M (2017). Closing the gender leadership gap: a multi-centre cross-country comparison of women in management and leadership in academic health centres in the European Union. Hum Resour Health.

[5] Deutscher Ärztinnenbund (2022). Medical women on top- update 2022.

[6] (n.d). Studierende der Medizin nach Geschlecht bis 2020/2021.

[7] Statistisches Bundesamt (DESTATIS) Bildung und Kultur- Studierende an Hochschulen -Vorbericht- 2022.

[8] (n.d). Promotionen in Deutschland: Statistik - Zahlen und Fakten zur Promotionsquote.

[9] (n.d). 192 300 Promovierende an deutschen Hochschulen im Jahr 2020.

[10] (n.d). Mehr Habilitationen von Frauen im Jahr 2020.

[11] Smith KS, Bakkensen JB, Hutchinson AP, Cheung EO, Thomas J, Grote V (2022). Knowledge of fertility and perspectives about family planning among female physicians. JAMA Netw Open.

[12] (n.d). 2021 U.S. Medical School Faculty.

