# Supplementary material for: Female physician and pregnancy- effect of the amended German maternity protection act on female doctors’ careers
Source: Innov Surg Sci. 2023 Mar 31;8(1):23–8. doi: 10.1515/iss-2022-0024 (PMC10576549; doi:10.1515/iss-2022-0024)
Supplement: Supplementary file 1 — Supplementary Material [file j_iss-2022-0024_suppl_001.pdf]

Reviewer Assessment

H. Raestrup et al.: Female physician and pregnancy- Effect of the amended German Maternity Protection Act on female doctors´ careers

Reviewers' Comments to Original Submission

Reviewer 1: Hidas, Christine

Date received: 06-Dec-2022
Reviewer recommendation: **Accept in present form**
Reviewer overall scoring: **Excellent**

Comments to author:
Very important topic, shows the reality in the hospital, the problems for the career of medical women on the basis of a purly legal law- this must be changed.

Reviewer 2: anonymous

Date received: 15-Dec-2022
Reviewer recommendation: **Accept in present form**
Reviewer overall scoring: **High**

Comments to author:
Important theme, good to show the effect of the "modernization" of the German Maternity Protection Act. The practical implementation doesn't entail any improvement of female participation....
